# Autoantibody-Mediated Depletion of IL-1RA in Still’s Disease and Potential Impact of IL-1 Targeting Therapies

**DOI:** 10.1007/s10875-023-01642-0

**Published:** 2024-01-17

**Authors:** Marie-Christin Hoffmann, Giulio Cavalli, Natalie Fadle, Eleonora Cantoni, Evi Regitz, Octavian Fleser, Philipp Klemm, Marina Zaks, Elisabeth Stöger, Corrado Campochiaro, Alessandro Tomelleri, Elena Baldissera, Jörg Thomas Bittenbring, Vincent Zimmer, Jochen Pfeifer, Yvan Fischer, Klaus-Dieter Preuss, Moritz Bewarder, Bernhard Thurner, Sabrina Fuehner, Dirk Foell, Lorenzo Dagna, Christoph Kessel, Lorenz Thurner

**Affiliations:** 1 José Carreras Center for Immuno- and Gene Therapy and Internal Medicine I, Saarland University Medical School, 66421 Homburg, Saarland, Germany; 2 Vita-Salute San Raffaele University, Milan, Italy; 3 Unit of Immunology, Rheumatology, Allergy, and Rare Diseases, IRCCS San Raffaele Scientific Institute, Vita-Salute San Raffaele University, 20132 Milan, Italy; 4 Department of Rheumatology, Immunology, Osteology and Physical Medicine, Justus-Liebig-University Gießen, Campus Kerckhoff, Bad Nauheim, Germany; 5 Department of Nephrology and Internal Intensive Care, Charité University Medicine Berlin, Campus Virchow Clinic, Berlin, Germany; 6 Evangelische Kliniken Essen-Mitte, Evangelische Huyssens-Stiftung Essen-Huttrop, Essen, Germany; 7 Department of Internal Medicine, Knappschaftsklinikum Saar, Püttlingen, Germany; 8 Department of Pediatric Cardiology, Saarland University, Homburg, Germany; 9 Institute of Physiology, Medical Faculty, RWTH Aachen, 52057 Aachen, Germany; 10 Medizinisches Versorgungszentrum, Mindelheim, Germany; 11 Department of Pediatric Rheumatology and Immunology, University Children’s Hospital Muenster, Münster, Germany

**Keywords:** Still’s disease, Systemic juvenile idiopathic arthritis (sJIA), Adult-onset Still’s disease (AOSD), Anti-IL-1Ra autoantibodies

## Abstract

**Background:**

Adult-onset Still’s disease (AOSD) and systemic juvenile idiopathic arthritis (sJIA) resemble a continuum of a rare, polygenic IL-1β-driven disease of unknown etiology.

**Objective:**

In the present study we sought to investigate a potential role of recently described autoantibodies neutralizing the interleukin-1(IL-1)-receptor antagonist (IL-1-Ra) in the pathogenesis of Still’s disease.

**Methods:**

Serum or plasma samples from Still’s disease patients (AOSD, *n* = 23; sJIA, *n* = 40) and autoimmune and/or inflammatory disease controls (*n* = 478) were analyzed for autoantibodies against progranulin (PGRN), IL-1Ra, IL-18 binding protein (IL-18BP), and IL-36Ra, as well as circulating IL-1Ra and IL-36Ra levels by ELISA. Biochemical analyses of plasma IL-1Ra were performed by native Western blots and isoelectric focusing. Functional activity of the autoantibodies was examined by an in vitro IL-1β-signaling reporter assay.

**Results:**

Anti-IL-1-Ra IgG were identified in 7 (27%) out of 29 Still’s disease patients, including 4/23 with AOSD and 3/6 with sJIA and coincided with a hyperphosphorylated isoform of endogenous IL-1Ra. Anti-IL-36Ra antibodies were found in 2 AOSD patients. No anti-PGRN or anti-IL-18BP antibodies were detected. Selective testing for anti-IL-1Ra antibodies in an independent cohort (sJIA, n = 34) identified 5 of 34 (14.7%) as seropositive. Collectively, 8/12 antibody-positive Still’s disease patients were either new-onset active disease or unresponsive to IL-1 blocking drugs. Autoantibody-seropositivity associated with decreased IL-1Ra plasma/serum levels. Seropositive plasma impaired in vitro IL-1Ra bioactivity, which could be reversed by anakinra or canakinumab treatment.

**Conclusion:**

Autoantibodies neutralizing IL-1Ra may represent a novel patho-mechanism in a subgroup of Still’s disease patients, which is sensitive to high-dose IL-1 blocking therapy.

**Supplementary Information:**

The online version contains supplementary material available at 10.1007/s10875-023-01642-0.

## Introduction

Systemic juvenile idiopathic arthritis (sJIA) and adult-onset Still’s disease (AOSD) are considered as polygenic autoinflammatory disorders. Recent gene expression data support the concept of a Still’s disease continuum that includes both a pediatric/juvenile (systemic juvenile idiopathic arthritis, sJIA) and adult onset (AOSD) form [1]. sJIA and AOSD are rare but are severe diseases with inflammatory manifestations that can affect multiple organs and can be complicated by macrophage activation syndrome (MAS) as a life-threatening hyperferritinemic cytokine storm condition. Following exclusion of infectious, autoimmune, or neoplastic causes, diagnosis is based on criteria established by Yamaguchi et al. [2]. On a serum/plasma biomarker level Still’s disease is further hallmarked by elevated levels of bioactive, free IL-18 (not complexed by IL-18 binding protein (IL-18BP)) [4]; as well as S100 proteins [6], sCD163 [7], IL-6, IL-8, IL-17, and TNF-α [8].

At present, excessive IL-1β release from Still’s disease patients’ immune cells and elevated levels in serum or plasma remains hard to detect, using state-of-the-art detection platforms. However, excellent response to IL-1 targeting therapies identifies both diseases as primarily driven by excessive IL-1β-signaling. Therapeutic IL-1 blockade either by substitution with recombinant human IL-1Ra (anakinra) [9] or by monoclonal antibody (canakinumab)-mediated neutralization of IL-1β [10] significantly improves disease outcomes in both AOSD and sJIA. Recent single cell expression and functional data further establish a prominent role of IL-1 in disease pathology [12–15].

Autoimmunity is not an established contributing factor in the pathogenesis of sJIA or AOSD, and sero-negativity for rheumatoid autoantibodies is an essential feature or hallmark of the final diagnosis. Yet, for sJIA, association with HLA-DRB1-11* and altered inflammatory T-lymphocyte subsets, in part driven by IL-1 signaling, were reported [14–17]. Beyond, recent data point to an enhanced development of inflammatory, antibody-production driving T helper cells and altered self-reactive IgG profiles in sJIA [18], supporting the concept that features adaptive immunity may drive Still’s disease progression [19–21]. For AOSD, certain HLA haplotypes were associated with the development and course of disease [22–25], and anecdotal reports suggest that individual patients with AOSD can potentially benefit from B-cell depletion [26].

Importantly, in both sJIA and AOSD, the specific molecular reasons for excessive IL-1 and potentially also IL-18 signaling are still poorly understood. In these lines, an association of polymorphisms in *IL1RN* (encoding IL-1Ra) identified in sJIA has been suggested to affect its expression in patients and thus result in an IL-1Ra:IL-1β imbalance and excessive IL-1β signaling [27]. While these data are still a matter of controversial discussion [28, 29], we recently reported on a new class of immune-modulatory autoantibodies targeting endogenous IL-1Ra [30] [31], as well as progranulin (PGRN), a receptor antagonist to TNFR1/TNFR2 [32] and DR3 [33], which we both observed in SARS-CoV-2 infection/inflammation–associated context. With respect to anti-PGRN autoantibodies, these data partly echoed previous observations in several autoimmune diseases including seronegative PsA and chronic inflammatory bowel diseases [34]. Of note, seropositivity for either anti-PGRN or anti-IL-1Ra antibodies was transient, associated with acute inflammation and coincided with unusual isoforms of the respective antigen due to hyperphosphorylation of a serine residue in position 81 (PGRN) [30] or threonine residue in position 111 (IL-1Ra)[31]. Importantly, anti-IL-1Ra antibodies depleted circulating IL-1Ra in plasma and impaired its bioactivity, thus promoting unrestricted IL-1β signaling [31].

Following these observations, we now hypothesize whether such immune-modulatory autoantibodies targeting IL-1Ra, IL-18BP and other anti-inflammatory mediators may also contribute to the pathogenesis of polygenic autoinflammatory disorders such as Still’s disease and may have been missed in previous investigations due to their transient nature.

## Patients and Methods

### Study Cohort

This retrospective study was approved by the local Ethical Review Boards (Ärztekammer des Saarlandes: 41/21; Muenster: 2015–670-fS; Milano: STS-CE 065/8) and conducted according to the Declaration of Helsinki. Blood plasma or serum samples were collected following written informed consent. In total, 63 Still’s disease patients (AOSD, *n* = 23; sJIA, *n* = 40; Table 1) were enrolled in the present study. Of one patient with initial diagnosis of AOSD complicated by MAS, one sample during acute inflammation and two follow-up samples were obtained.

**Table 1:** Patient characteristics

		sJIA (*n* = 40) (cohort 1 and 2)	sJIA Pos for IL-1RA-Ab (*n* = 8)	sJIA Neg for IL-1RA-Ab (*n* = 32)	AOSD (*n* = 23)	AOSD Pos for IL-1RA-Ab (*n* = 4)	AOSD Neg for IL-1RA-Ab (*n* = 19)	Control auto-immune diseases (*n* = 447)	UC (*n* = 50)	CD (*n* = 50)	SLE (*n* = 50)	RA (*n* = 100)	AAV (*n* = 50)	PMR (*n* = 47)	PSA (*n* = 100)
Age (y)	Median (range)	13.4 (4.2–60)	15.4	13.3	45 (21–76)	58.5	43	55 (12–96)	39.5 (19–80)	40 (12–70)	46.5 (18–80)	63 (28–96)	56 (16–79)	72 (40–85)	54.5 (24–81)
Sex															
Male		23 (57.5%)	4 (50%)	19 (59.4%)	7 (30.43%)	1 (25%)	6 (31.6%)	138 (30.87%)	24 (48%)	25 (50%)	7 (14%)	20 (20%)	16 (32%)	16 (34%)	30 (30%)
Female		17 (42.5%)	4 (50%)	13 (40.6%)	16 (69.57%)	3 (75%)	13 (68.4%)	309 (69.13%)	26 (52%)	25 (50%)	43 (86%)	80 (80%)	34 (68%)	31 (65%)	70 (70%)
Ethnicity															
Caucasian		14 (35%)	3 (37.5)	11 (34.3%)	22 (95.65%)	4 (100%)	18 (94.7%)								
Arab		4 (10%)	2 (25%)	2 (6.3%)	0 (0%)	0 (0%)	0 (0%)								
Latin		0 (0%)	0 (0%)	0 (0%)	1 (4.35%)	0 (0%)	1 (5.3)								
Unknown		22 (55%)	5 (62.5%)	19 (59.4)	0 (0%)	0 (0%)	0 (0%)	447 (100%)							
Age at onset (y)	Median (range)	11.0 (0.1–17.7)	6.35	11.0	30 (18–70)	52	29	nk	nk	nk	nk	nk	nk	nk	nk
Disease activity															
Active		22 (55%) (nk = 3; 7.5%)	5 (62.5%)	17 (53.1%) (nk = 3; (9.4%))	5 (21.74%)	1 (25%)	4 (21%)	nk	nk	nk	nk	nk	nk	nk	nk
Inactive		15 (37.5%)	3 (37.5%)	12 (37.5%)	18 (78.26%)	3 (75%)	15 (79%)								
History of MAS															
+		6 (15%) (nk = 6%; 15%)	2 (25%), (nk = 1, (12.5%))	4 (12.5%); (nk = 5;(15.6%))	4 (17.39%)	2 (50%)	2 (10.5%)	nk	nk	nk	nk	nk	nk	nk	nk
−		28 (70%)	5 (62.5%)	23 (71.9%)	19 (82.61%)	2 (50%)	17 (89.5%)								
History of SARS-CoV-2 infection															
+		0 (0%) (nk = 22; 55%)	-		1 (4.35%)	0 (0%)	1 (5.3%)	0 (0%)	0 (0%)	0 (0%)	0 (0%)	0 (0%)	0 (0%)	0 (0%)	0 (0%)
−		18 (45%)	5 (62.5%); (nk = 3; (37.5%))	13 (40.6%); (nk = 19; (59.4%))	22 (95.65%)	4 (100%)	18 (94.7%)	447 (100%)	50 (100%)	50 (100%)	50 (100%)	100 (100%)	50 (100%)	47 (100%)	50 (100%)
Anakinra															
+		18 (45%) (nk = 1; 2.5%)	4 (50%)	13 (40.6%); (nk = 1, (3.1%))	17 (73.91%)	4 (100%)	13 (68.4%)		(nk = 50; 100%)	(nk = 50; 100%)	(nk = 50; 100%)	(nk = 100; 100%)	(nk = 50; 100%)	(nk = 47; 100%)	(nk = 100; 100%)
−		21 (52.5%)	4 (50%)	18 (56.3%)	6 (26.09%)	0 (0%)	6 (31.6%)								
Canakinumab															
+		4 (10%) (nk = 1; 2.5%)	1 (12.5%)	2 (6.3%); (nk = 1 (3.1%))	9 (39.13%)	2 (50%)	7 (36.8%)		(nk = 50; 100%)	(nk = 50; 100%)	(nk = 50; 100%)	(nk = 100; 100%)	(nk = 50; 100%)	(nk = 47; 100%)	(nk = 100; 100%)
−		35 (87.5%)	7 (87.5%)	29 (90.6%)	14 (60.87%)	2 (50%)	12 (63.2%)								
Other DMARDs															
+		13 (32.5%) (nk = 1; 2.5%)	4 (50%)	9 (%); (nk = 1 (%))	14 (60.87%) (nk = 2; 8.7%)	nk = 1	14 (73.7%); nk = 1 (5.3%)		(nk = 50; 100%)	(nk = 50; 100%)	(nk = 50; 100%)	(nk = 100; 100%)	(nk = 50; 100%)	(nk = 47; 100%)	(nk = 100; 100%)
−		26 (65%)	4 (50%)	22 (%)	7 (30.43%)	3 (75%)	4 (21.1%)								
IL-1Ra-Abs															
+		8 (20%)	8 (100%)	0 (0%)	4 (17.39%)	4 (100%)	0 (0%)	6 (1.34%)	2 (4%)	1 (2%)	1 (2%)	1 (1%)	0 (0%)	1 (2.13%)	0 (0%)
−		32 (80%)	0 (0%)	32 (100%)	19 (82.61%)	0 (0%)	19 (100%)	441 (98.66%)	48 (96%)	49 (48%)	49 (48%)	99 (99%)	50 (100%)	46 (97.87%)	100 (100%)
IL-1Ra plasma level pg/ml								nd							
Media		1586.5	788.5	1655	1327	916.5	1358								
Mean		1516.1	640.6	1734.9	1282	875.8	1368								
Range		185–2381	185–859	1097–2381	235–2011	235–1435	722–2011								

*UC* ulcerative colitis, *CD* Crohn’s disease, *SLE* systemic lupus erythematosus, *RA* rheumatoid arthritis, *AAV* ANCA-associated vasculitis, *PMR* polymyalgia rheumatica, *PSA* psoriatic arthritis, + yes, − no, *nk* not known, *nd* not done

Out of the entire Still’s disease study cohort (*n* = 63, Table 1), *n* = 29 patients (cohort 1 AOSD, *n* = 23; sJIA, *n* = 6; Table S1) were included for an initial screening approach aiming at a broader analysis for autoantibodies targeting anti-inflammatory mediators. All study cohort patient samples were collected at the Department of Internal Medicine I of the Saarland University Hospital (Homburg/Saar, Germany), the unit of Immunology, Rheumatology, Allergy, and rare diseases, IRCCS San Raffaele Scientific (Milan, Italy).

To further test for prevalence of anti-IL-1Ra antibodies in Still’s disease, we further enrolled samples obtained from *n* = 34 sJIA and *n* = 1 AOSD patients (Table S2) collected at the Department of Pediatric Rheumatology and Immunology at University Children’s Hospital Muenster (cohort 2). Collectively, for both cohorts 1 and cohort 2, we aimed at identifying serum and plasma samples as closest to disease onset as possible. Yet, due to the retrospective nature of the study, the interval between clinical disease onset and biosamples included in this study is not standardized.

As study controls, plasma samples of various inflammatory autoimmune diseases (ANCA-associated vasculitides, *n* = 50; systemic lupus erythematosus, *n* = 50; rheumatoid arthritis, *n* = 100; polymyalgia rheumatic, *n* = 47; psoriatic arthritis, *n* = 100; Crohn’s disease, *n* = 50; ulcerative colitis, *n* = 50) were included, all collected at the department of Internal Medicine I and II at Saarland University Hospital (Homburg/Saar, Germany).

### ELISA for Autoantibodies Against PGRN, IL-1Ra, IL-18BP, and IL-36Ra

Respective ELISA were performed as described in the supplemental methods section.

### Western Blot and Isoelectric Focusing of IL-1Ra, PGRN, and IL-36Ra

Western blot and isoelectric focusing of IL-1Ra and IL-36Ra were performed as described in the supplemental methods section.

### ELISA for Plasma Level Determination of IL-1Ra or IL-36Ra

IL-1Ra plasma levels were determined with a commercially available ELISA kit (Invitrogen/ThermoFisher #BMS2080) and IL-36Ra plasma level with a commercially available ELISA kit (Adipogen, #AG-46B-0006-KI01) according to the manufacturer’s instructions.

### IL-1 Signaling Reporter Assay

The IL-1 signaling reporter assay using HEK-Blue™ IL-1β reporter cells was done as detailed in the supplemental methods section.

### Analysis of Inflammatory Patients’ Mediators in Serum or Plasma

Multiplexed analysis of serum or plasma proteins was performed as described in the supplemental methods section.

### Statistics

Differences in proportions of IL-1Ra-autoantibody-positivity between AOSD/sJIA and autoimmune disease were compared by the Fisher exact test. Association between two categorical variables was tested with the two-tailed Fisher exact test. Distributions of IL-1 Ra plasma determined by ELISA were tested for normality by the Shapiro–Wilk test. Means of normally distributed plasma levels of IL-1Ra between patient subgroups with or without IL-1Ra-Abs were then compared by two-tailed non-paired *t*-test. For the analysis comparing seropositive with seronegative subgroups, patients with AOSD and sJIA were combined. No correction for multiple analysis was done.

## Results

### Autoantibodies Targeting Anti-Inflammatory Mediators in Still’s Disease

**Fig. 1:**
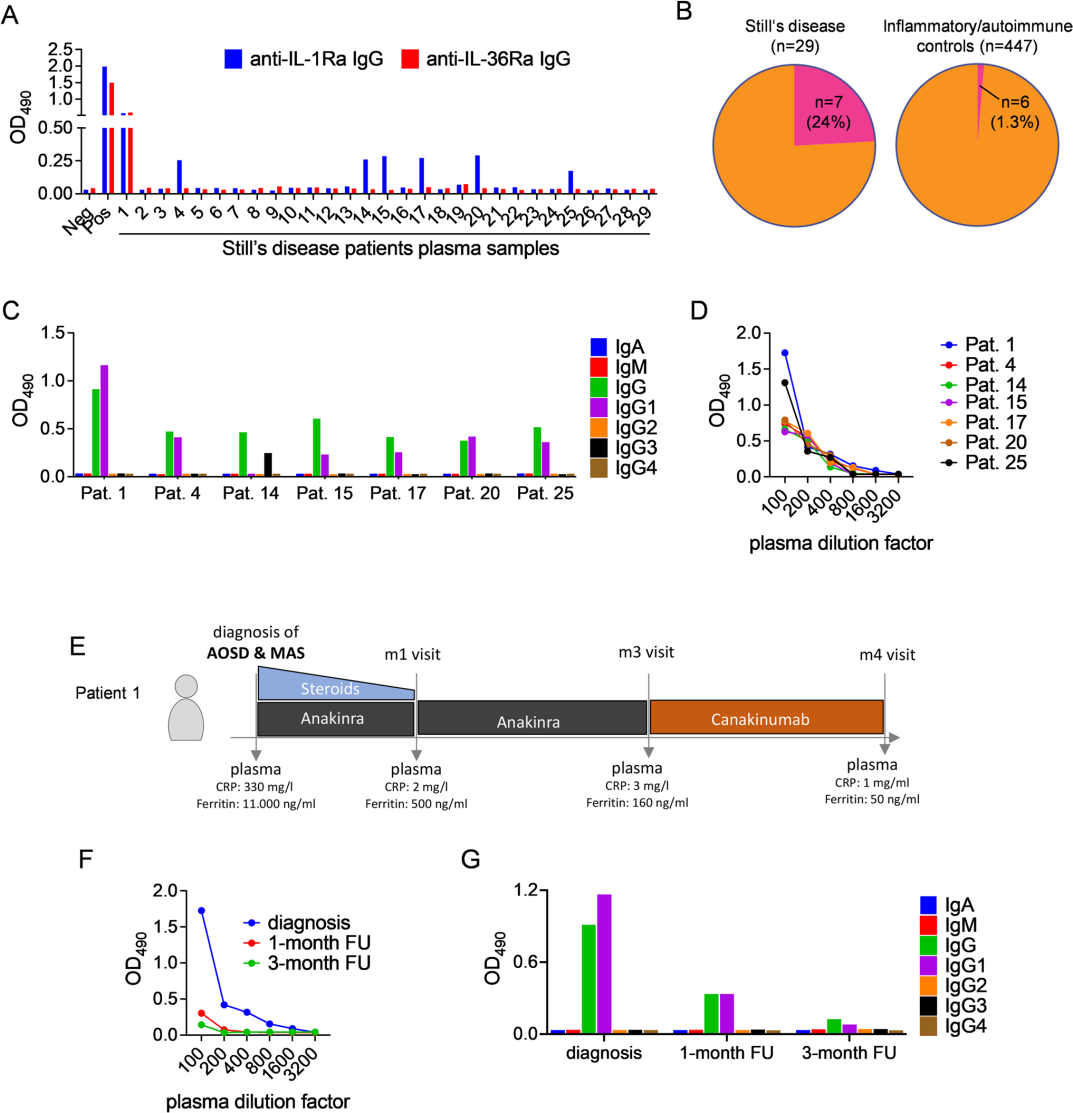
Anti-IL-1Ra and anti-IL-36Ra antibodies in Still’s disease. **A** Anti-IL-1Ra and anti-IL-36Ra antibodies in Still’s disease patients’ plasma (sJIA, patient 13, 14, 16, 17, 18, 20) were detected by ELISA. **B** Seven (24%) out of 29 patients vs. six (1.3%) out of 447 inflammatory and autoimmune controls revealed anti-IL-1Ra antibodies. **C**, **D** IL-1Ra reactive Ig type and IgG subclasses as well as titers of anti-IL-1Ra IgG in patients’ plasma were tested by ELISA. **E** Schematic clinical course and treatment regime of a newly diagnosed AOSD patient (patient 1) with macrophage activation syndrome. **F**, **G** Anti-IL-1Ra IgG titers in diagnosis and follow-up plasma samples of patient 1 as well as IL-1Ra reactive Ig type and IgG subclasses were tested by ELISA. All data are presented as means and represent 2 replicates

**Fig. 2:**
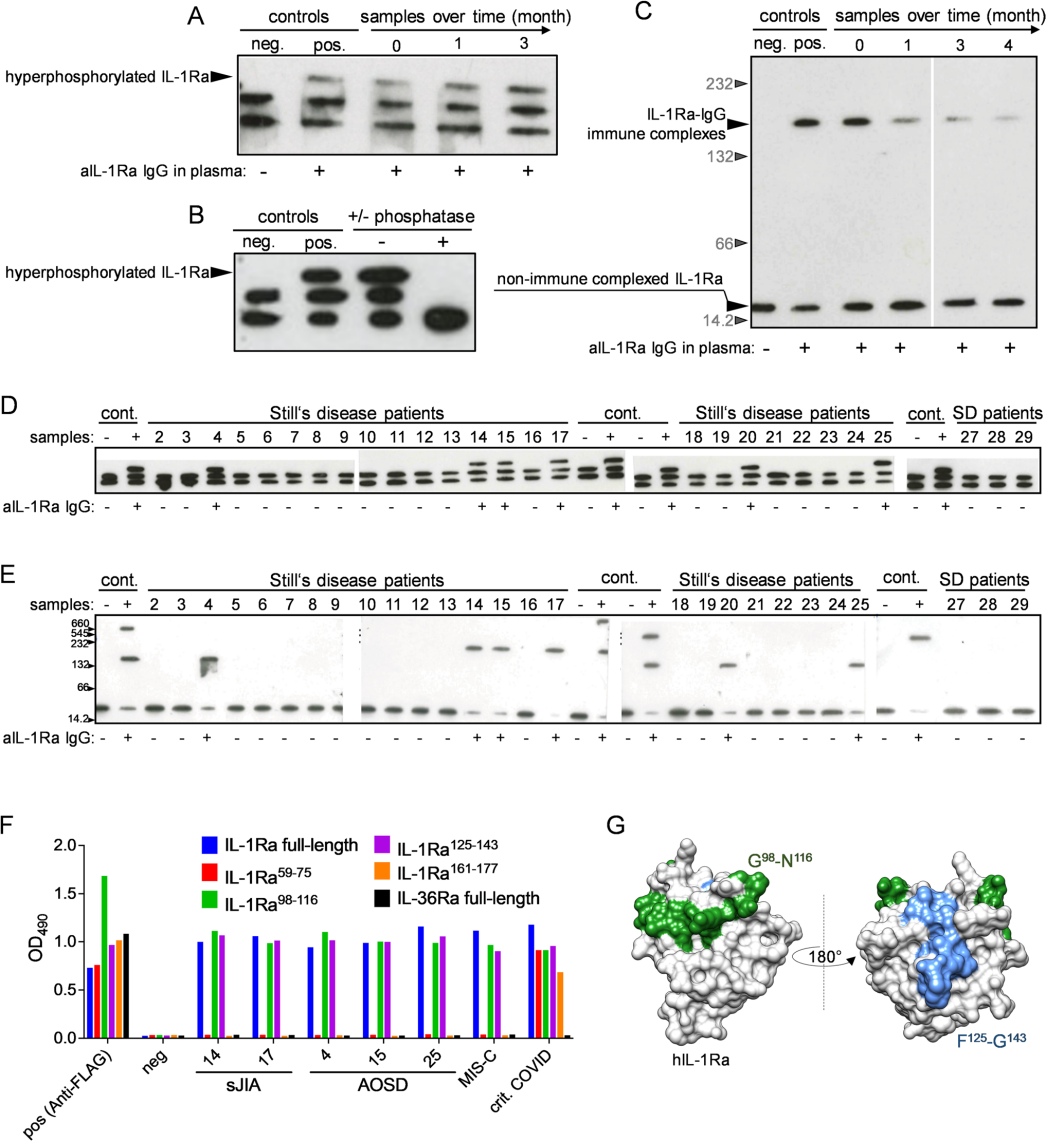
Anti-IL-1Ra antibodies in Still’s disease associate with IL-1Ra hyperphosphorylation and recognize similar epitopes as in MIS-C. **A** Isoelectric focusing (IEF) of plasma IL-1Ra in diagnosis and follow-up samples of patient 1. Plasma samples previously tested positive or negative for anti-IL-1Ra antibodies were used as positive or negative control. **B** IEF with or without alkaline phosphatase treatment of IL-1-Ra in plasma of patient 1. Positive and negative controls as described above. **A**, **B** Protein bands in IEF representing hyperphosphorylated IL-1Ra are highlighted. **C** Western blot of native PAGE for IL-1RA:IgG immune complexes in diagnosis and follow-up plasma samples of patient 1. Positive and negative controls as described above. Protein bands representing immune complexed and non-immune complexed “free” IL-1Ra are highlighted. **D**, **E** IEF of plasma IL-1Ra (**D**) or Western blot of native PAGE for IL-1RA:IgG immune complexes (**E**) in 29 Still’s disease patients. Positive and negative controls as well as respective protein bands have been described above. Seropositivity for anti-IL-1Ra IgG according to ELISA (Fig. 1A) is indicated by + / − . **F** Approximative epitope mapping of anti-IL-1Ra antibodies in selected sJIA and AOSD plasma samples compared to MIS-C and COVID-19 using IL-1Ra derived synthetic peptides. Full-length IL-36 was used as negative control. Data are presented as means. **G** Visualization of the identified antigenic determinants of IL-1Ra reactive IgG in patients’ plasma on the solvent exposed surface of human IL-1Ra (1ilr.pdb)

Among Still’s disease patients in cohort 1 (*n* = 29; AOSD, *n* = 23; sJIA, *n* = 6), 7 (24%; AOSD, *n* = 4; sJIA, *n* = 3) were tested seropositive for antibodies binding to IL-1Ra (Fig. 1A, B). Among inflammatory/autoimmune controls (*n* = 447), anti-IL-1Ra antibodies were found in 6 out of 447 investigated patients (1.3%; Fig. 1B, Figure S1). In all autoantibody-positive Still’s disease patients or controls, anti-IL-1Ra antibodies belonged predominantly to the IgG1 subclass (Fig. 1C, Figure S1H). In Still’s disease patients, anti-IL-1Ra antibody titers ranged between 1:200 and 1:400 (Fig. 1D). One patient with new-onset AOSD also complicated by MAS tested positive for both anti-IL-1Ra and anti-IL-36Ra antibodies (patient 1, Fig. 1A). Of this patient, four longitudinal samples covering both acute inflammation as well as remission were available (Fig. 1E). At initial presentation, anti-IL-1Ra antibody titers ranged up to 1:800 and antibodies belonged to the IgG1 subclass exclusively (Fig. 1F, G). Following initiation of anakinra treatment, autoantibody titers dropped to 1:100 (Fig. 1F). After 3 months of daily anakinra injections and partial remission, treatment was switched to targeted IL-1β neutralization using canakinumab. At 3 months follow-up visit, the anti-IL-1Ra antibody titers were still detectable at a 1:100 titer (Fig. 1F), while a 4-month-follow-up plasma sample enrolled in protein biochemical analysis still revealed immune complexed IL-1Ra in Western blot (Fig. 2C). Among all patients investigated in cohort 1, no antibodies specific for PGRN or IL-18BP were detected (Figure S2).

### Hyperphosphorylated Atypical IL-1Ra Isoforms, Immune Complexed IL-1Ra and Anti-IL-1Ra Epitope Specificity in Still’s Disease

In our previous analysis we observed seropositivity for anti-IL-1Ra antibodies to coincide with hyperphosphorylated isoforms of IL-1Ra in plasma [30, 31, 36]. Isoelectric focusing (IEF) performed on total protein from plasma of the AOSD patient with new-onset disease complicated by MAS with samples during acute inflammation and remission (Fig. 1E) revealed the presence of an additional, negatively charged IL-1Ra protein band at the time of presentation (Fig. 2A). Pretreatment with alkaline phosphatase prior to IEF resulted in disappearance of both the normally occurring second as well as the atypical additional third IL-1Ra isoform, thus indicating a hyperphosphorylation (Fig. 2B). In the follow-up samples available with this patient, hyperphosphorylated IL-1Ra in plasma was still detectable at the 3- and 4-month-follow-up visits after initial diagnosis and MAS, and coinciding with seropositivity (Figs. 1F and 2A). In native Western blots of total plasma protein of this patient, we further observed an additional protein band representing IgG-bound IL-1Ra (Fig. 2C). Signal intensity of this band was weakening alongside with decreasing anti-IL-1Ra IgG titers in plasma (Figs. 1F and 2C).

Beyond, hyperphosphorylated IL-1Ra was observed in all six anti-IL-1Ra autoantibody seropositive Still’s disease patients in cohort 2 (*n* = 29) but not in any of the seronegative individuals (Fig. 2D). In native Western blots and coinciding with hyperphosphorylated IL-1Ra, we observed both a weakened protein band of free IL-1Ra (approx. 17 kDa) as well as a band representing IgG-bound IL-1Ra in all seropositive Still’s disease patients (Fig. 2E).

Throughout, it needs to be emphasized that in contrast to Western blots of native gradient gels and sample preparation in native, non-reducing buffer (Fig. 2C and E), the pretreatment for IEFs results in a dissociation of immune complexes in serum or plasma samples (Fig. 2A, B, and D). Therefore, protein bands in the IEF analysis engulf both formerly autoantibody-bound as well as free IL-1Ra and thus cannot reflect an antibody-mediated protein depletion. In consequence, in IEFs, there is no difference in IL-1Ra protein band intensities.

Beyond these biochemical analysis, in a comparative epitope mapping approach on recombinant IL-1Ra fragments, we observed anti-Il-1Ra IgG in plasma of both seropositive sJIA as well as AOSD patients to preferentially bind to peptides spanning amino acids to a region spanning G^98^-N^116^ and F^125^-G^143^. This echoes the epitope specificity of anti-IL-1Ra antibodies in multisystem inflammatory syndrome in children (MIS-C) but differs from those observed in severe COVID-19 (Fig. 2F, G).

### Autoantibody-Mediated IL-1Ra Depletion Across Different Disease States and Sensitivity to IL-1 Blocking Therapies

**Fig. 3:**
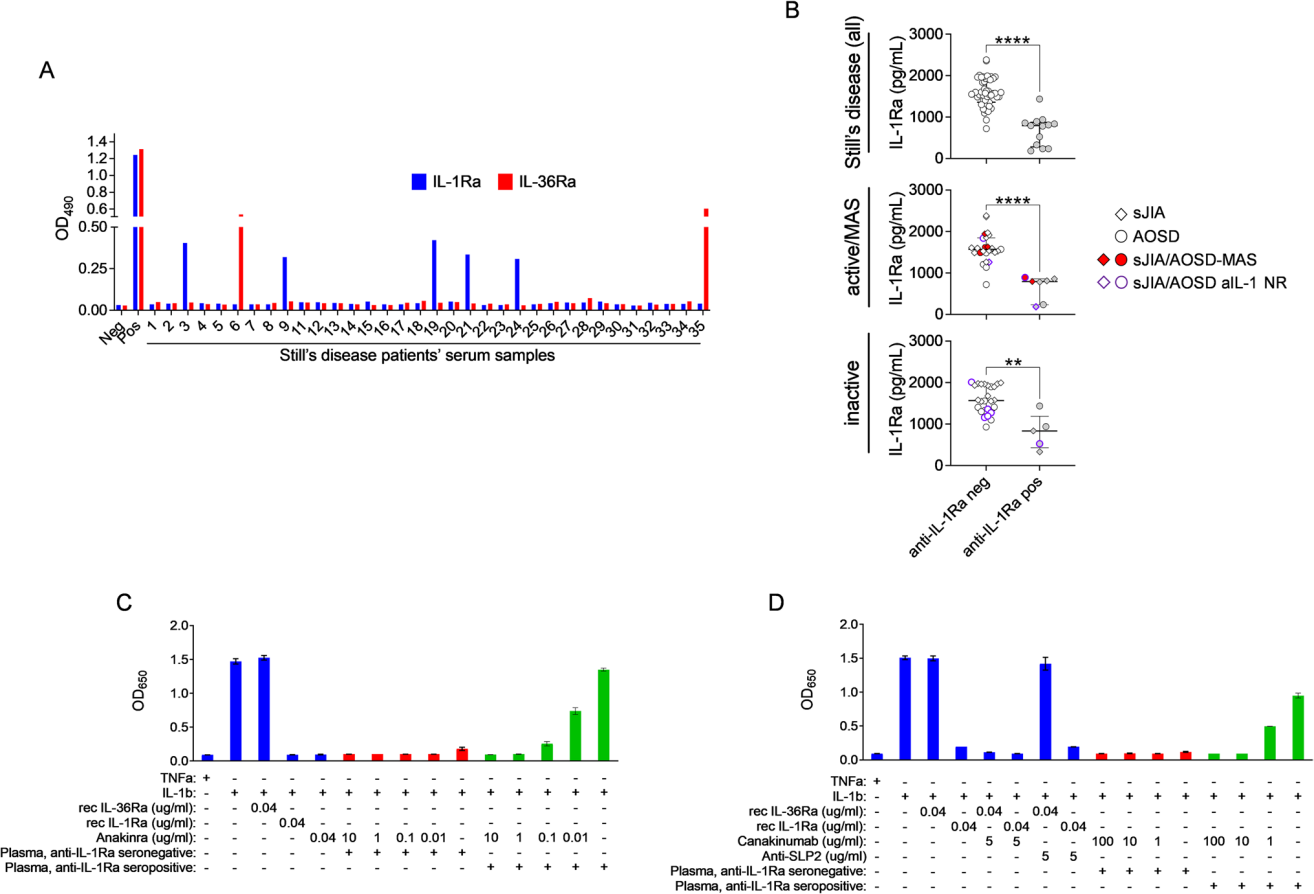
Autoantibody mediated IL-1Ra depletion across different disease states and sensitivity to IL-1 blocking therapies. **A** Anti-IL-1Ra and anti-IL-36Ra antibodies as determined by ELISA in sera of an independent Still’s disease patients’ cohort (AOSD, patient 35). **B** IL-1Ra levels in anti-IL-1Ra IgG positive or negative Still’s disease plasma or serum samples, including strafication for underlying disease activity and response to aIL-1 treatment. Data are represented as individual values and were analyzed by Mann–Whitney *U* test. Lines indicate median and error bars the interquartile range. **p ≤ 0.01; ****p ≤ 0.0001. MAS, macrophage activation syndrome, aIL-1 NR, anti-IL-1 therapy non-responder. **C**, **D** IL-1b signaling assay using HEK IL-1 reporter cells to evaluate the efficacy of anakinra (**C**) or canakinumab (**D**) to override the IL-1Ra depleting effect of anti-IL-1Ra positive Still’s disease plasma. In this assay cohort 1 plasma samples 1 (anti-IL-1Ra titer 1:800) and 6 (seronegative) were used at a dilution of 1:20. TNFa, IL-36Ra, and a monoclonal anti-stomatin-like protein 2 (SLP2) antibody (targeting an assay-irrelevant protein) were used as negative controls in these assays. Data are presented as mean + / − SD of triplicates

In order to level the number of analyzed AOSD and sJIA patients in our study and to further balance for an eventual center bias in terms of sample quality and collection procedures as well as sample matrix (serum vs. plasma), we enrolled a second cohort of predominantly sJIA patients (*n* = 34; AOSD, *n* = 1) and analyzed respective serum samples for anti-IL-1Ra as well as anti-IL-36Ra antibodies. In this cohort we identified anti-IL-1Ra antibodies in 5 out of 34 sJIA patients (15%) and observed anti-IL-36Ra antibodies in one sJIA and 1 AOSD patient (Fig. 3A). As additional disease controls, we also tested patients with clinically (fever of unknown origin, FUO, *n* = 8) or genetically defined autoinflammatory syndromes (familial Mediterranean fever, FMF, *n* = 3; cryopyrin-associated periodic syndrome, CAPS, *n* = 2), which are in part primarily driven by excessive IL-1b expression and signaling and thus present with an immunopathology similar to that of Still’s disease. Here, we identified only very low levels of IL-1Ra reactive IgG and IgM antibodies (titer of 1:100) in a single FUO patient, which also coincided with a decrease in serum IL-1Ra levels. All other patients were tested negative for anti-IL-1Ra antibodies (Figure S3).

As observed among patients in cohort 1 (Fig. 2D, E), hyperphosphorylated IL-1Ra was found in all anti-IL-1Ra autoantibody seropositive Still’s disease patients in cohort 2 (*n* = 29) but not seronegative individuals (Figure S4A). In Western blots of native gradient PAGE, we observed both a weakened protein band of free IL-1Ra (approx. 17 kDa) as well as a band representing IgG-bound IL-1Ra in all seropositive Still’s disease patients (Figure S4B). Importantly, in these analysis, we identified 1 patient (patient 24 of cohort 2), which tested positive for anti-IL-1Ra IgG in both ELISA (Fig. 3A) as well as Western blot (Figure S4B), but we could not observe hyperphosphorylation of endogenous IL-1Ra by IEF (Figure S4A). Similarly, we observed hyperphosphorylated IL-1Ra and immune complexed IL-1Ra with a single FUO patient (Figure S5A, B) who was tested positive for anti-IL-1Ra antibodies by ELISA (Figure S3).

In contrast to changes in endogenous IL-1Ra observed in anti-IL-1Ra seropositive Still’s disease patients, no such changes in protein charge or additional isoforms could be observed with IL-36Ra in case of seropositivity for anti-IL-36Ra antibodies (Figure S6A). Yet, in native Western blots, we also observed IL-36Ra:IgG immune complexes in those patients’ serum samples (Figure S6B), which also tested seropositive in ELISA for anti-IL-36Ra IgG (Fig. 3A).

Next, we analyzed the entire Still’s disease study cohort (*n* = 63; AOSD, *n* = 24; sJIA, *n* = 39) for IL-1Ra plasma or serum levels and observed those significantly decreased (unpaired *t* test for parametric data, two tailed *p* < 0.0001; *t* = 7.985; df = 61) in anti-IL-1Ra seropositive patients (mean 719.1 pg/ml, SD 350.3 pg/ml), when compared to seronegative cases (mean 1598 pg/ml, SD 341.7 pg/ml) (Fig. 3B, upper panel).

Beyond, anti-IL-1Ra antibodies could be detected in both active/MAS patients (7/32, 22%; Fig. 3B, center panel), as well as inactive Still’s disease patients (5/31, 16%; Fig. 3B, lower panel) and appeared not associated with non-response to IL-1 targeting therapy (where this information was available, Tables S1and S2) or other clinical disease features (Table S3). Among already treated patients, the detection of IL-1Ra-Abs was not associated with previous anakinra treatment (*p* > 0.9999; two-sided Fisher’s exact test; Table S4).

Moreover, we could not observe an obvious link with inflammatory cytokine levels in anti-IL-1Ra seropositive vs. seronegative sJIA and AOSD patients (Figure S7). In seropositive sJIA patients, IL-6 levels were elevated by trend (*p* = 0.08) compared to anti-IL-1Ra seronegative patients. No such tendency was observed among patients with AOSD. No differences could be observed with respect to IL-18 serum/plasma levels in both sJIA and AOSD. Soluble VCAM-1 concentrations were significantly decreased in sera of anti-IL-1Ra seropositive compared to seronegative sJIA patients, but only within overall range of healthy control levels (Figure S7A).

Employing high sensitivity proteomics using proximity extension assay on a very limited number of patients’ samples confirmed elevation in IL-6 and a few other markers in seropositive vs. seronegative patients (Figure S7B–D).

Next to depletion of circulating IL-1Ra, our previous studies [30, 36, 37] indicated an anti-IL-1Ra IgG mediated impairment of IL-1Ra bioactivity, resulting in unopposed IL-1 signaling [30, 31, 36]. Similarly, we also observed antibodies in Still’s disease plasma to disturb IL-1Ra function and facilitate unrestricted IL-1β signaling. Beyond, we also tested the sensitivity of this IgG-mediated detrimental impact on IL-1Ra function to IL-1 targeting therapies. In such lines, we investigated the potential effect of increasing concentrations of anakinra or canakinumab on IL-1β signaling in the presence of anti-IL-1-Ra IgG from diluted plasma. In these assays on an IL-1β signaling reporter cell line, both anakinra and canakinumab in concentrations above 1 µg/ml (anakinra) or 10 µg/ml (canakinumab) ameliorated the net proinflammatory impact anti-IL-1Ra antibodies in patient’s plasma (Fig. 3C, D, and Figure S8).

## Discussion

Despite in part substantial scientific progress, the pathogenesis of many polygenic autoinflammatory diseases such as systemic juvenile idiopathic arthritis (sJIA, Still’s disease) as well as its counterpart in adults (adult-onset still’s disease, AOSD) still remains poorly understood. Both sJIA and AOSD are currently considered to represent a continuum of one and the same disease [37]. Both conditions frequently respond well to IL-1β/IL-1R1-blocking drugs, which argues for excessive/dysregulated IL-1β signaling to play a central role in Still’s disease pathology. Lack of understanding regarding specific disease triggers presents an eminent blind spot in the pathophysiologic understanding of polygenic autoinflammation. Yet, several observational data suggest infections to play an essential role as initial triggers of disease. In this respect our recent observations on an infectious/inflammatory context, [30, 31, 36] triggering a transient IL-1Ra depleting autoantibody response is highly intriguing as it may indicate a potential starting point of the IL-1-driven pathology in Still’s disease.

Here, we report on the occurrence of neutralizing autoantibodies targeting IL-1Ra in Still’s disease patients. Collectively, we identified 18.75% (12/64; sJIA 20.51%, 8/39; AOSD 12.5%, 3/24) of patients as seropositive for anti-IL-1Ra antibodies. In contrast, those were identified in only 1.3% (6/447) autoimmune or inflammatory controls. Apart, autoantibodies targeting IL-36Ra were found in two AOSD patients, while anti-PGRN or anti-IL-18BP antibodies were detected in none of the investigated individuals.

Seropositivity for anti-IL-1Ra antibodies in Still’s disease in all but one patient (patient 24, cohort 2) coincided with hyperphosphorylation of endogenous IL-1Ra, as already observed in our previous studies in context of SARS-CoV-2 infection or mRNA vaccination [30, 31, 36]. Approximative peptide-based epitope mapping of anti-IL-1Ra IgG in both sJIA and AOSD indicated similar antigenic determinants as recognized in MIS-C, but different from COVID-19. Of note, the identified epitopes in sJIA, AOSD, and MIS-C (G^98^-N^116^ and F^125^-G^143^) overlapped with those associated with anti-IL-1Ra antibodies recently described in context of IgG4-related disease (F^100^-G^119^ and M^136^-E^152^; Jarrell et al., 2022). Anti-IL-1Ra antibodies in Still’s disease depleted IL-1Ra in serum and plasma; however, this depletion was less pronounced as observed in previous studies in SARS-CoV-2 context [30, 31, 36], likely due to lower anti-IL-1Ra titers generally observed in Still’s disease.

Despite these intriguing (mechanistic) findings, at present, we cannot clearly associate seropositivity for anti-IL-1Ra IgG in Still’s disease with clinical disease activity. Frequency of seropositive patients presenting with active disease (including present or history of MAS) compared to inactive disease appears elevated by trend (22% vs. 16%, chi-square *p* value 0.56). This observation is further bolstered by data we reported earlier on 10 sJIA patients in long-standing remission, none of which revealed IL-1Ra targeting antibodies [31]. Next to poor association with disease activity, we also could not link presence of anti-IL-1Ra antibodies with non-response to anakinra treatment, a recombinant truncated form of human IL-1Ra expressed in *E. coli* that is administered by daily subcutaneous injection. Of patients where we had such information available, only four reported non-responders (two of those with active disease) tested seropositive for anti-IL-1Ra IgG in contemporaneous serum/plasma samples, whereas seven reported non-responders were tested seronegative. Collectively, we believe that in the present study, the transient occurrence of both the autoantibodies and IL-1Ra hyperphosphorylation renders analysis as discussed above complicated. The hyperphosphorylated IL-1Ra isoform potentially precedes autoantibody appearance, as suggested from our previous data of longitudinal samples demonstrating a disappearance of the hyperhosphorylated IL-1Ra isoform while IL-1Ra targeting antibodies were still present [31]. This may also be seen with patient 24 of cohort 2 in the present study. Thus, in order to better understand a link of autoantibody occurrence with clinical disease activity or response to treatment, this requires biosampling synchronized with disease on-set, flare, or non-response to therapy, ideally including longitudinal follow-up.

Beyond, we cannot identify a significant association of seropositivity for anti-IL-1Ra antibodies with Still’s disease hallmark cytokine levels such as IL-6 or IL-18. The lack of such associations can be due to overall low frequency of seropositive vs. seronegative patients or to impact of auto-antibody-mediated IL-1Ra depletion at tissue rather than whole blood level. In these lines, investigations in other context point towards little to no impact of therapeutic IL-1 blockade on an inflammatory whole blood signature (our own yet unpublished data), and stimulation of whole blood with recombinant IL-1β demonstrates surprisingly weak overall inflammatory impact by this cytokine [38, 39]. In contrast, we and others have demonstrated strong effect of anti-IL-1 treatment on inflammatory activation of, i.e., endothelial cells [40] and also associate this with decreased shedding of VCAM-1 due to persistent inflammatory endothelial activation [40], as also observed in the present data. Further, our recent observations on an association of anti-IL-1Ra antibodies with the influx of CD3 + and CD68 + cells into the myocardium may also argue for a tissue directed rather than systemic impact of these auto-antibodies [36]. Nonetheless, in the present study, the overall lack of such associations hampers the interpretation on the impact of the observed autoantibodies on Still’s disease pathology.

Non-apparent correlation of anti-IL-1Ra antibodies with clinical activity (markers) may be reminiscent of the role of anti-neutrophil cytoplasmic antibodies (ANCAs) in systemic vasculitis. In many cases, ANCA-titers only correlate weakly with disease activity [42], while in vitro evidence and preclinical models suggest a prominent proinflammatory impact [42, 43][44], and it is thus still debatable whether these antibodies represent an epiphenomenon or can be considered critical drivers of disease [45].

Systemic JIA patients are frequently treated by daily subcutaneous injections of anakinra. In case of clinical benefit from the IL-1 blocking therapeutic approach, treatment can be switched to canakinumab, an IL-1β neutralizing monoclonal antibody. While IL-1 targeting therapies represent the gold-standard of treatment in sJIA and are also applied first-line [46], in AOSD, they are frequently introduced later in the disease course, and guidelines still recommend the use of drugs such as methotrexate or calcineurin inhibitors (i.e., cyclosporin) [47]. Importantly, in our study, both anakinra and canakinumab at concentrations in the range of peak plasma or serum levels of both drugs according to reported pharmacokinetic data (anakinra (2 mg/kg/day), approx. 2 µg/ml [48]; canakinumab (4.5 mg/kg), approx. 40 µg/mL [49]) could outcompete the inhibitory effect of anti-IL-1Ra antibodies on IL-1Ra bioactivity in Still’s disease plasma. Importantly, these data suggest standard treatment protocols to possibly override eventual detrimental auto-antibody-mediated effects on disease pathology.

Collectively, our study findings require to be interpreted in the light of several limitations. [1] Our study is retrospective and thus analyzes a very heterogenous cohort of patients with respect to clinical disease courses and underlying treatments, with no standardized sampling protocols. [2] The overall number of identified seropositive patients is small and this—as discussed above—limits interpretations. [3] The cause or trigger of IL-1Ra hyperphosphorylation still remains unclear, and we were unable to associate anti-IL-1Ra seropositivity with, i.e., HLA as these data were only available for a fraction of sJIA patients.

Yet, the present study is the first to report on anti-IL-1Ra autoantibodies in a polygenic autoinflammatory disease. Interestingly, our molecular findings on the association with IL-1Ra hyperphosphorylation, antibody specificity, and impact on IL-1Ra plasma/serum levels and bioactivity do phenocopy earlier observations in SARS-CoV-2 context. We speculate that our findings may indicate evidence for autoimmune events in a subset of (early) sJIA and AOSD pathology that may destabilize IL-1:IL-1Ra balance and could thus result in long-standing or recurring episodes of autoinflammation. Importantly, we demonstrate that state-of-the-art IL-1-targeting therapies can override the negative auto-antibody impact on IL-1Ra bioactivity.

### Supplementary Information

Below is the link to the electronic supplementary material.

Supplementary file1 (DOCX 3057 KB)

## Data Availability

All data required to evaluate the conclusions in the paper are present in the manuscript or its appendix. Further information on the study protocol or de-identified datasets generated and analyzed within this publication are available from the corresponding authors on reasonable request.
