# Supplementary material for: Autoantibody-Mediated Depletion of IL-1RA in Still’s Disease and Potential Impact of IL-1 Targeting Therapies
Source: J Clin Immunol. 2024 Jan 17;44(2):45. doi: 10.1007/s10875-023-01642-0 (PMC10794369; doi:10.1007/s10875-023-01642-0)
Supplement: Supplementary file 1 — Supplementary file1 (DOCX 3057 KB) [file 10875_2023_1642_MOESM1_ESM.docx]

**Supplement**

**Autoantibody mediated depletion of IL-1RA in Still’s Disease and potential impact of IL-1 targeting therapies**

Marie-Christin Hoffmann^a^, Giulio Cavalli^bc^, Natalie Fadle^a^, Eleonora Cantoni^b^, Evi Regitz^a^, Octavian Fleser^a^, Philipp Klemm^d^, Marina Zaks^e^, Elisabeth Stöger^f^, Corrado Campochiaro^b^, Alessandro Tomelleri^b^, Elena Baldissera^b^, Jörg Thomas Bittenbring^a^, Vincent Zimmer^i^, Jochen Pfeifer^j^, Lorenzo Dagna^b^, Yvan Fischer^k^, Klaus- Dieter Preuss^a^, Moritz Bewarder^a^, Bernhard Thurner^l^, Sabrina Fühner^b^, Dirk Foell^b^, Lorenzo Dagna^bc^, Christoph Kessel^b**^, Lorenz Thurner^a**^

** shared senior authors

^a^ José Carreras Center for Immuno- and Gene Therapy and Internal Medicine I, Saarland University Medical School, Homburg/Saar, Germany

^b^ Vita-Salute San Raffaele University, Milan, Italy

^c^ Unit of Immunology, Rheumatology, Allergy, and Rare Diseases, IRCCS San Raffaele Scientific Institute, Vita-Salute San Raffaele University, 20132 Milan, Italy

^d^ Department of Rheumatology, Immunology, Osteology and Physical Medicine, Campus Kerckhoff, Justus-Liebig-University Gießen, Bad Nauheim, Germany

^e^ Department of Nephrology and Internal Intensive Care, Charité University Medicine Berlin, Campus Virchow Clinic, Berlin, Germany

^f^ Evangelische Kliniken Essen-Mitte, Evangelische Huyssens-Stiftung Essen-Huttrop, Essen, Germany

^g^ Department of Internal Medicine V - Pulmonology, Allergology and Critical Care Medicine, Saarland University, Homburg, Germany

^h^ Department of Internal Medicine II, Saarland University Medical School, Homburg/Saar, Germany

^i^ Department of Internal Medicine, Knappschaftsklinikum Saar, Püttlingen, Germany

^j^ Department of Pediatric Cardiology, Saarland University, Homburg, Germany

^k^ Institute of Physiology, Medical Faculty, RWTH Aachen, D-52057 Aachen, Germany

^l^ Medizinisches Versorgungszentrum Mindelheim, Germany

^b^ Department of Pediatric Rheumatology and Immunology, University Children’s Hospital Muenster

Corresponding author:

Lorenz Thurner, MD

Dept. of Internal Medicine I and José-Carreras-Center for Immuno- and Gene Therapy

Saarland University Medical School

D-66421 Homburg/Saar, Germany

e-mail: [lorenz.thurner@uks.eu](mailto:lorenz.thurner@uks.eu);

Phone: +49-6841-1615362

**Supplementary Methods**

**ELISA for autoantibodies against PGRN, IL-1-Ra, IL-18BP and IL-36-Ra**

The ELISA for autoantibodies was performed as previously described (1). In short, the antigens were obtained using the coding sequences of the *GRN* gene encoding PGRN, isoform 1 precursor of *IL1RN* were recombinantly expressed with a C-terminal FLAG-tag in HEK293 cells under the control of a cytomegalovirus promoter (pSFI). Total cell extracts were prepared and bound to Nunc MaxiSorp plates (eBioscience, Frankfurt, Germany) precoated with murine anti-FLAG mAb at a dilution of 1:2,500 (v/v; Sigma-Aldrich, Munich, Germany) at 4°C overnight. After blocking with 1.5% (w/v) gelatin in Tris-buffered saline (TBS) and washing steps with TBS with Triton X-100, the individual plasma samples were diluted 1:100. ELISA was performed according to standard protocols with the following Abs: biotinylated goat antihuman heavy and light chain immunoglobulin G (IgG) at a dilution of 1:2,500 (Dianova, Hamburg, Germany); subclass-specific sheep antihuman IgG1, IgG2, IgG3 and IgG4 (Binding Site Group, Birmingham, UK) at dilutions of 1:5,000; goat antihuman IgM (Dianova) at a dilution of 1:2,500; or goat antihuman IgA (Dianova) at a dilution of 1:2,500. Following this step, corresponding biotinylated secondary Abs were used for immunoassays carried out to detect IgG subclasses and IgM. Peroxidase-labelled streptavidin (Roche Applied Science, Indianapolis, IN, USA) was used at a dilution of 1:50,000. As a cut-off for positivity, the average of the optical density (OD) of the negative samples plus three standard deviations was applied. To narrow down the epitope region of the IL-1-Ra-Abs, full-length IL-1-Ra, following fragments thereof: amino acid 59-75, 98-116, 125-143 and 161-177, and as a control antigen full-length IL-36-Ra were recombinantly expressed with a C-terminal FLAG tag in HEK293 cells under the control of a cytomegalovirus promoter (pSFI).

**Western blot, isoelectric focusing of IL-1-Ra and IL-36-Ra**

Isoelectric focusing (IEF) and Western blotting (including native Western blotting with non-reducing sample pretreatment and gradient (4-20%) native gels without SDS and native buffer) were performed. For isoelectric focusing plasma samples were stored at −20°C until use. Equal volumes of sample and loading buffer were mixed. Samples were analyzed by isoelectric focusing (IEF) on a gel with a fixed pH gradient (pH 3-10) according to the manufacturer's instructions (Novex pH 3-10; Invitrogen), followed by an immunoblot screening. After plasma samples were separated by IEF or PAGE electrophoresis, the proteins were transferred to a Immobilon-P polyvinylidene difluoride membrane (Millipore Immobilon) by semidry blotting.

The membrane was blocked overnight at 4°C in TBST/milk buffer [10% (vol/vol) milk in 10mM Tris/HCl, pH 7.5, 150mM NaCl, and 0.1% (vol/vol) Tween 20], washed, and incubated for 1 hour at room temperature with serum in TBST (paraprotein-containing serum from patients at a dilution of 1:10^8^ and from controls at a dilution of 1:10^3^). Blocking was done overnight at 4°C in 10% non-fat dry milk (weight/volume). After 3 washings in TBST, the membranes were incubated for 1 hour at room temperature with either rabbit Anti human IL1RA (antibodies.online #ABIN2856394) at dilution of 1:2000 or after stripping with rabbit Anti human IL-36RA (GeneTex #108421)at dilution of 1:2000 in TBST, subsequently incubated for 1hour at room temperature with anti-rabbit IgG HRP-conjugate, followed by washing in TBST, and finally by detection with the use of Pharmacia enhanced chemiluminescence system (General Electric). Plasma samples were analyzed for IL-1-Ra isoforms. Plasma from IL-1-Ra-Ab-positive patient was treated with alkaline phosphatase as previously described using FastAP thermo-sensitive alkaline phosphatase (Fermentas/VWR, Darmstadt, Germany) (2). Stripping of membranes was performed with glycine buffer (0.2M glycine pH 2.2; 0.1% SDS; 1X Tween 20).

**IL-1 signaling reporter assay**

For IL-1ß assay HEK-Blue™ IL-1β reporter cells (Invivogen, #hkb-il1bv2) were used, which react specifically to IL-1ß and IL-1α by induction of NF-κB/AP-1, leading to expression of a secreted embryonic alkaline phosphatase (SEAP) reporter. Anakinra at 0.01 to 10µg/mL, recombinant IL-1-Ra at 40ng/mL (Biozol, #PPT-AF-2000-01RA), recombinant IL-36Ra at 40ng/mL, canakinumab 1 to 100µg/ml and Anti-SLP2 IgG antibody(2) at 5 µg/ml were used.

In addition, plasma diluted 1:20 of patient with AOSD with (AOSD #I) or without IL-1Ra-Abs (AOSD#29) were preincubated for 2h at room temperature with IL-1Ra, anakinra or canakinumab. Subsequently, these compounds were added with either IL-1ß (Biozol,# PPT-200-01B) or TNF-α (Biozol, #PPT-300-01A) both at 2ng/mL in 100µl DMEM to 2x10E4 HEK-Blue™ IL-1β reporter cells per well and incubated overnight at 37°C. Thereafter, 180 µL of each supernatant was transferred, 20µl QUANTI-BlueTM (Invivogen, #rep-qbs) was added and SEAP activity was measured at OD of 655nm. Experiments were performed in triplicate.

**Multiplexed bead array assay**

Reagents for multiplexed quantification of IL-6, IL-18 and sVCAM-1 were purchased from R&D Systems (Minneapolis, OH, USA). Reagents and sera or cell culture supernatants were prepared according to the manufacturer’s instructions (R&D Systems). Data acquisition and analysis was performed on a MAGPIX instrument (Merck Millipore, Darmstadt, Germany) using xPONENT v4.2 software (Luminex).

**Proteomic analysis**

Serum proteomics was performed using proximity extension assays (PEA) on the Olink^®^ Target 96 platform, using Target 96 Inflammation and Target 96 Cardiovascular III panels. All samples were analyzed by Olink Proteomics, Uppsala, Sweden. Data were reported in normalized protein expression values (npx). NPX is an arbitrary unit in a Log2 scale calculated from inverted, normalized Ct values. All assay validation data are available on the Olink website ([www.olink.com](http://www.olink.com)).

**Supplementary Table S1: Patient characteristics, Still’s disease cohort 1**

| **Patient number** | **Gender (m/f)** | **Age [years]** | **Ethnicity** | **Age at onset of AOSD** | **Active disease** | **MAS** | **History of SARS-CoV-2** | **Anakinra** | **Canakinumab** | **Other DMARD** | **Glucosteroids** | **B-cell-depleted** | **IL-1-Ra-Ab** | **Titer of IL-1-Ra-Ab** | **Ig class of IL-1-Ra-Ab** | **Hyperphosphorylated IL-1Ra** | **IL-1-Ra plasma level pg/ml** |
| --- | --- | --- | --- | --- | --- | --- | --- | --- | --- | --- | --- | --- | --- | --- | --- | --- | --- |
| 1 | f | 49 | caucasian | 49 | + | + | - | Started (after blood sample was taken) | Later in the course |  | + | - | + | 1:800 | IgG1 | + | 235 |
| 2 | m | 33 | caucasian | 25 | - | - | - | - | - | MTX (2015 -ongoing) | Stop 04/20 |  | - | na | na | - | 1327 |
| 3 | f | 43 | caucasian | 30 | - | - | - | 03/07-05/07 (inefficacy) | - | ADA (2007 -ongoing) | ongoing |  | - | na | na | - | 1358 |
| 4 | f | 76 | caucasian | 58 | - | + | - | Secondary inefficiency two years ago;  05/05-05/19 (inefficacy) | Started, after inefficacy of anakinra (05/19 -ongoing) | - | ongoing |  | + | 1:800 | IgG1 | + | 893 |
| 5 | f | 35 | caucasian | 29 | - | - | - | - | - | MTX (06/16-ongoing); TCZ (12/16-ongoing) | Stop (05/18) |  | - | na | na | - | 1404 |
| 6 | m | 34 | caucasian | 28 | - | - | - | 12/18-05/19 (inefficacy) | 05/19 - ongoing | - | Stop (06/20) |  | - | na | na | - | 1274 |
| 7 | f | 58 | caucasian | 50 | + | - | - | 04/19-01/20 | 01/20-01/21 | - | - |  | - | na | na | - | 1137 |
| 8 | f | 72 | caucasian | 70 | - | - | - | 04/19-10/19 (intolerance) | 11/19 - ongoing | - | Stop (01/21) |  | - | na | na | - | 1195 |
| 9 | m | 52 | latin | 49 | - | - | - | 07/19 - ongoing | - | CsA(12/18-ongoing) | Stop (06/21) |  | - | na | na | - | 1301 |
| 10 | f | 21 | caucasian | 21 | + | - | - | - | 05/21 - ongoing | MTX (03/21 -ongoing) | Stop (06/21) |  | - | na | na | - | 722 |
| 11 | f | 27 | caucasian | 25 | + | - | - | 05/21 - ongoing | 09/20 - 05/21 (inefficacy) | MTX (03/19 -ongoing) | ongoing |  | - | na | na | - | 1838 |
| 12 | m | 25 | caucasian | 18 | - | + | - | 05/14 - ongoing | - | TOC (05/13-06/13, inefficacy) | Stop (05/18) |  | - | na | na | - | 1490 |
| 13 | f | 60 | caucasian | 6 | - | - | - | - | - | CZP (01/18-12/20, inefficacy); ETN (12/20 -ongoing) | Stop (2010) |  | - | na | na | - | 1898 |
| 14 | m | 16 | caucasian | 3 | + | - | - | Was shortly on anakinra, stopped because lack of efficacy and anaphylactic reaction | - | CsA, MTX | + |  | + | 1:400 | IgG1 | + | 185 |
| 15 | m | 41 | caucasian | 21 | - | - | - | Optimal disease control under anakinra (2006 -ongoing) | - | - | Stop (02/07) |  | + | 1:400 | IgG1 | + | 1435 |
| 16 | f | 33 | caucasian | 2 | - | - | - | never | - | IFX (2006-2009, inefficacy); GOL (2009-2010, inefficacy); CZP (2010-2011, inefficacy); ADA (2013-ongoing) | Stop (2000) |  | - | na | na | - | 1097 |
| 17 | f | 9 | caucasian | 6 | - | - | - | +, discontinued for inefficacy | +, discontinued for inefficacy | MTX | + |  | + | 1:800 | IgG1 | + | 526 |
| 18 | m | 17 | caucasian | 6 | + | - | - | +, discontinued for inefficacy | - | TOC, MTX; currently on Baricitinib | + |  | - | na | na | - | 1261 |
| 19 | f | 48 | caucasian | 26 | - | - | - | 12/06 - 06/07 (inefficacy) | - | TOC (04/12-ongoing) | Stop (06/13) |  | - | na | na | - | 1158 |
| 20 | m | 18 | caucasian | 17 | - | - | - | - | - | - | Stop (07/21) |  | + | 1:400 | IgG1 | + | 331 |
| 21 | f | 45 | caucasian | 20 | - | - | - | 11/06-ongoing | - | - | Stop (08/09) |  | - | na | na | - | 1558 |
| 22 | f | 52 | caucasian | 41 | - | - | - | - | - | MTX (01/21-ongoing) | ongoing |  | - | na | na | - | 927 |
| 23 | f | 42 | caucasian | 39 | - | - | + (12/20) | - | - | MTX (08/19-ongoing) | Stop (02/20) |  | - | na | na | - | 1409 |
| 24 | m | 51 | caucasian | 31 | - | - | - | 04/08-12/13 (inefficacy) | - | TOC (12/13-ongoing) | Stop (12/17) |  | - | na | na | - | 2011 |
| 25 | f | 68 | caucasian | 55 | - | - | - | Optimal disease control under anakinra (04/09-ongoing) | - | - | Stop (04/18) |  | + | 1:400 | IgG1 | + | 940 |
| 26 | f | 30 | caucasian | 29 | - | + | - | 12/20-ongoing | - | CsA (12/20-ongoing) | ongoing |  | - | na | na | - | 1629 |
| 27 | m | 63 | caucasian | 47 | - | - | - | - | - | Cyclophosphamid, Leflunomid, Rituximab, MTX | Stop | + | - | na | na | - | 1570 |
| 28 | f | 26 | caucasian | 26 | + | - | - | Initiated (after blood sample) | + |  | + | - | - | na | na | - | 1209 |
| 29 | f | 72 | caucasian | 65 | - | - | - | Discontinued (01/17), restarted and ongoing since 05/19 | discontinued (10/17) | MTX | - | - | - | na | na | - | 1487 |

ANA: Anakinra, ETC: Etanercept; CAN: Canakinumab; CsA: Ciclosporin A; IFX: infliximab; MTX: methotrexate; TOC: Tocilizumab; +: yes; -: no; nk: not known; nd: not done; na: not applicable

**Supplementary Table S2: Patient characteristics, Still’s disease cohort 2**

| **Patient number** | **Gender (m/f)** | **Age [years]** | **Ethnicity** | **Age at onset** | **Active disease** | **MAS** | **History of SARS-CoV-2** | **Anakinra** | **Canakinumab** | **Other DMARD** | **Glucosteroids** | **IL-1-Ra-Ab** | **IL-1-Ra plasma level pg/ml** | **Titer of IL-1-Ra-Ab** | **Ig class of IL-1-Ra-Ab** | **Hyperphosphorylated IL-1Ra** | **IL-36-Ra-Ab** | **IL-36-Ra plasma level ng/ml** | **Titer of IL-36-Ra-Ab** | **Ig class of IL-36-Ra-Ab** |
| --- | --- | --- | --- | --- | --- | --- | --- | --- | --- | --- | --- | --- | --- | --- | --- | --- | --- | --- | --- | --- |
| 1 | m | 12.3 | Caucasian | 11.9 | + | nk | - | - | - | - | + | - | 1855 | na | na | - | - | nd | na | na |
| 2 | m | 9 | Caucasian | 8.4 | + | nk | - | - | - | - | + | - | 1487 | na | na | - | - | nd | na | na |
| 3 | f | 6.3 | Arab | 1.3 | + | nk | - | + | - | MTX | Later in the course | + | 783 | 1:400 | IgG1 | + | - | nd | na | na |
| 4 | f | 18.9 | Caucasian | 1.6 | + | nk | - | - | - | TOC | - | - | 1538 | na | na | - | - | 77.1 | na | na |
| 5 | f | 14.1 | Caucasian | 13.5 | + | - | - | - | - | MTX | + | - | 1527 | na | na | - | - | 91.4 | na | na |
| 6 | m | 11.8 | Caucasian | 11.8 | + | nk | - | + | - | - | + | - | 1915 | na | na | - | + | 5.7 | 1:800 | IgG1 |
| 7 | f | 5.3 | Arab | 0.5 | - | + | - | 2 short periods of ANA; latest 2y before blood sample | started next visit after blood sample | CsA | + | - | 1941 | na | na | - | - | nd | na | na |
| 8 | f | 17.8 | Caucasian | 1.2 | + | - | - | - | - | MTX, ETC | - | - | 2356 | na | na | - | - | nd | na | na |
| 9 | f | 14.8 | Arab | 3.4 | + | - | - | - | - | MTX, TOC | - | + | 859 | 1:400 | IgG1 | + | - | nd | na | na |
| 11 | m | 13.5 | Caucasian | 12.9 | (+) | nk | - |  |  |  |  | - | 1576 | na | na | - | - | nd | na | na |
| 12 | m | 14.8 | Arab | 13.8 | + | - | - | relapse after switch ANA -> CAN | + | - | - | - | 2381 | na | na | - | - | nd | na | na |
| 13 | f | 18.2 | Caucasian | 0.1 | + | - | - | - | - | - | + | - | 1606 | na | na | - | - | nd | na | na |
| 14 | f | 12.4 | nk | 11.4 | - | - |  | - | - | - | - | - | 1977 | na | na | - | - | nd | na | na |
| 15 | m | 13.3 | nk | 13.1 | + | - |  | + | - | - | - | - | 1599 | na | na | - | - | nd | na | na |
| 16 | m | 13.3 | nk | 13.1 | + | - |  | + | - | - | - | - | 1264 | na | na | - | - | nd | na | na |
| 17 | m | 14.0 | nk | 13.8 | nk | - |  | - | - | - | - | - | 1679 | na | na | - | - | nd | na | na |
| 18 | f | 5.9 | nk | 2.0 | + | - |  | - | - | - | - | - | 1496 | na | na | - | - | nd | na | Na |
| 19 | f | 17.0 | nk | 16.4 | + | + |  | - | - | - | - | + | 794 | 1:400 | IgG1 | + | - | nd | na | na |
| 20 | m | 16.1 | nk | 13.8 | nk | status after |  | - | - | - | - | - | 1547 | na | na | - | - | nd | na | na |
| 21 | m | 7.3 | nk | 6.7 | + | status after |  | - | - | - | - | + | 811 | 1:400 | IgG1 | + | - | nd | na | na |
| 22 | m | 20.6 | nk | 13.8 | - | - |  | - | - | - | - | - | 1551 | na | na | - | - | nd | na | na |
| 23 | m | 8.0 | nk | 6.7 | - | status after |  | + | - | + | + | - | 1935 | na | na | - | - | nd | na | na |
| 24 | m | 16.7 | nk | 16.3 | - | - |  | + | - | - | - | + | 836 | 1:800 | IgG1 + IgG2 | - | - | nd | na | na |
| 25 | m | 11.1 | nk | 11.0 | + | - |  | + | - | - | - | - | 1971 | na | na | - | - | nd | na | na |
| 26 | m | 11.1 | nk | 11.0 | + | + |  | - | - | - | - | - | 1631 | na | na | - | - | nd | na | na |
| 27 | m | 10.2 | nk | 11.0 | + | - |  | + | - | - | - | - | 1528 | na | na | - | - | nd | na | na |
| 28 | f | 4.2 | nk | 3.3 | - | - |  | + | - | - | - | - | 1971 | na | na | - | - | nd | na | na |
| 29 | m | 17.6 | nk | 13.1 | - | - |  | - | - | - | - | - | 1995 | na | na | - | - | nd | na | na |
| 30 | f | 7.7 | nk | 1.2 | - | - |  | + | - | + | - | - | 1578 | na | na | - | - | nd | na | na |
| 31 | f | 19.9 | nk | 16.4 | - | - |  | - | + | - | - | - | 1935 | na | na | - | - | nd | na | na |
| 32 | f | 7.3 | nk | 6.5 | - | - |  | + | - | - | - | - | 1899 | na | na | - | - | 87.0 | na | na |
| 33 | m | 12.2 | nk | 11.0 | - | - |  | - | - | - | - | - | 1963 | na | na | - | - | 96.4 | na | na |
| 34 | m | 5.5 | nk | 4.4 | + | - |  | + | - | - | - | - | 1595 | na | na | - | - | 85.8 | na | na |
| 35 | m | 17.8 | nk | 17.7 | nk | - |  | + | - | - | - | - | 1965 | na | na | - | + | 6.3 | 1:400 | IgG1 |

None of the patients of the validation cohort was B-cell-depleted. ANA: Anakinra, ETC: Etanercept; CAN: Canakinumab; CsA: Ciclosporin A; MTX: methotrexate; TOC: Tocilizumab; +: yes; -: no; nk: not known; nd: not done; na: not applicable

**Supplementary Table S3. Clinical characteristics, Still’s disease cohort 2**

| **Patient number** | **Fever** | **Arthritis** | **Rash** | **ANA status** | **anti-IL-1Ra** |
| --- | --- | --- | --- | --- | --- |
| 1 | no | no | no | n/a | - |
| 2 | no | no | n/a | - | - |
| 3 | n/a | yes | no | n/a | + |
| 4 | no | yes | no | n/a | - |
| 5 | n/a | n/a | n/a | - | - |
| 6 | yes | yes | no | + | - |
| 7 | no | no | yes | - | - |
| 8 | no | yes | no | - | - |
| 9 | no | yes | yes | n/a | + |
| 11 | n/a | n/a | no | - | - |
| 12 | no | no | yes | n/a | - |
| 13 | no | yes | no | n/a | - |
| 14 | n/a | n/a | n/a | + | - |
| 15 | yes | yes | n/a | ++ | - |
| 16 | yes | yes | n/a | ++ | - |
| 17 | yes | n/a | yes | ++ | - |
| 18 | yes | yes | yes | n/a | - |
| 19 | yes | n/a | yes | - | + |
| 20 | no | no | no | ++ | - |
| 21 | yes | n/a | yes | + | + |
| 22 | no | no | no | ++ | - |
| 23 | no | yes | yes | + | - |
| 24 | no | no | no | - | + |
| 25 | no | yes | no | n/a | - |
| 26 | no | yes | no | n/a | - |
| 27 | no | yes | no | n/a | - |
| 28 | no | no | no | - | - |
| 29 | no | n/a | n/a | ++ | - |
| 30 | no | n/a | n/a | + | - |
| 31 | no | no | yes | - | - |
| 32 | no | no | yes | - | - |
| 33 | no | no | no | n/a | - |
| 34 | yes | yes | yes | + | - |
| 35 | no | n/a | no | n/a | - |

**Supplementary Table S4. Fisher’s exact test, prior vs. no prior exposure to anakinra**

| Data analyzed | pos IL-1Ra-Abs | neg IL-1Ra-Abs | Total |
| --- | --- | --- | --- |
| prior Anakinra | 7 | 26 | 33 |
| never Anakinra | 6 | 23 | 29 |
| Total | 13 | 49 | 62 |

| P value and statistical significance |  |
| --- | --- |
| Test | Fisher's exact test |
| P value | >0,9999 |
| P value summary | ns |
| One- or two-sided | Two-sided |
| Statistically significant (P < 0.05)? | No |

**Figure S1. Analysis of inflammatory and autoimmune controls.** A-G) Raw ELISA data (OD_490_) analyzing plasma samples from patients with ANCA associated vasculitides (A), ulcerative colitis (B), Crohn’s disease (C), polymyalgia rheumatica (D), psoriatic arthritis (E), rheumatoid arthritis (F), and systemic lupus erythematosus (G) for anti-IL-1Ra antibodies (IgG). Positive assay controls are indicated by green, anti-IL-1Ra IgG positive patients’ samples are highlighted by red triangles. (H) Ig classes and IgG subclasses of IL-1-Ra-Abs in seropositive inflammatory/autoimmune patients were determined by ELISA. (I) Isoelectric focusing (IEF) of IL-1Ra in plasma of inflammatory/autoimmune patients with or without anti-IL-1Ra antibodies. Seropositivity for anti-IL-1Ra IgG according to ELISA (A-H) is indicated by +/-.

**Figure S2. Anti-PGRN and anti-IL-18bp antibodies in Still’s disease patients’ plasma (cohort 1)**. Raw ELISA data (OD_490_) analyzing plasma samples from Still’s disease patients for anti-progranulin (PGRN) as well as anti-IL-18 binding protein (IL-18bp) antibodies (IgG).

**Figure S3. Prevalence of anti-IL-1Ra antibodies in autoinflammatory diseases with similar immunopathology compared to Still’s disease.** ELISA data (OD_490_) analyzing serum samples of fever of unknown origin (FUO), familial mediterranean fever (FMF) and cryopyrin associated periodic syndrome (CAPS) patients for anti-IL-1Ra antibodies (OD_490_) and serum levels of IL-1Ra (pg/mL). All patients were sampled during active disease and off medication. *MEFV*-mutation status is indicated with FMF patients (hom: homozygous; het: heterozygous).


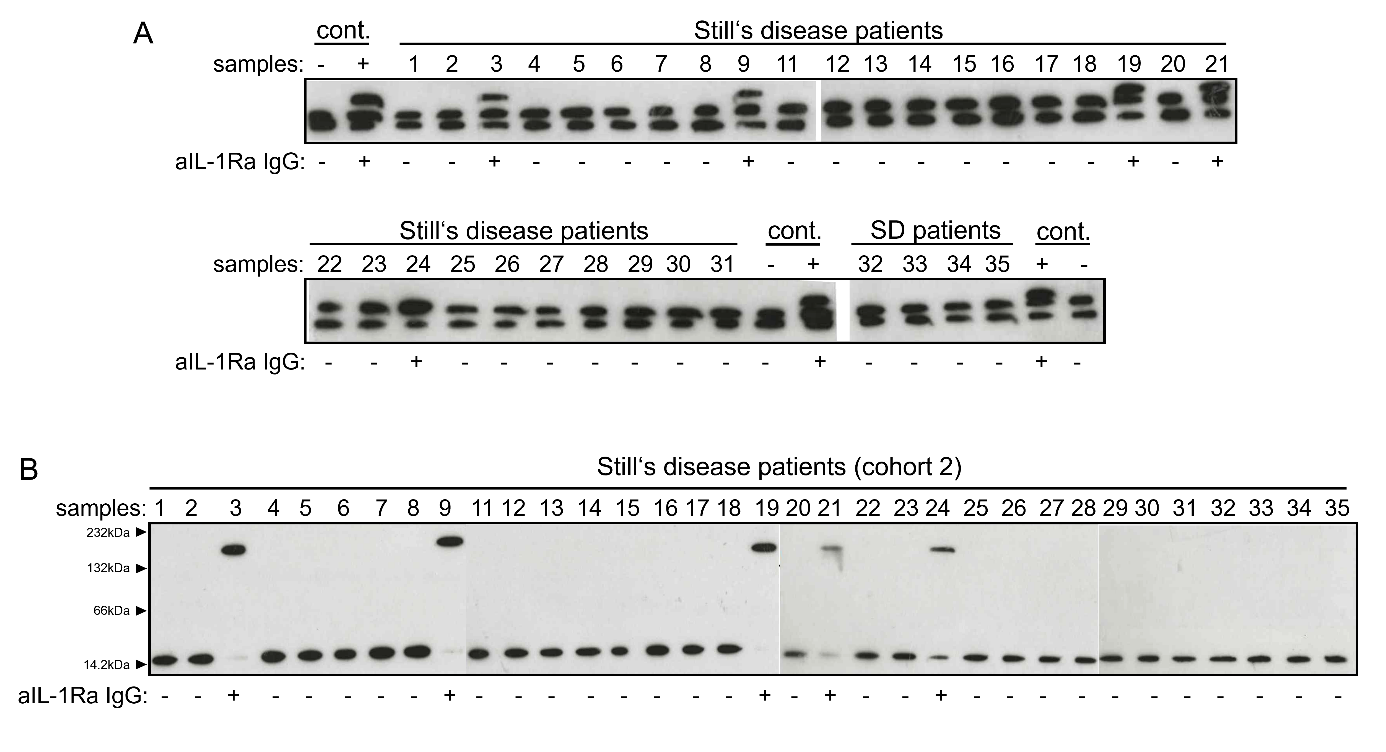


^§^

**Figure S4. IL-1Ra hyperphosphorylation and immune complexes in Still’s disease patients’ plasma (cohort 2).** (A) Isoelectric focusing (IEF) of plasma IL-1Ra or (B) Western blot of native gradient PAGE for IL-1RA:IgG immune complexes in 35 Still’s disease patients (cohort 2). Plasma samples previously tested positive or negative for anti-IL-1Ra antibodies were used as positive or negative control. Seropositivity for anti-IL-1Ra IgG according to ELISA (**Figure 3A**) is indicated by +/-. ^§^Patient 24 revealed no hyperphosphorylation of endogenous IL-1Ra by IEF (**A**) but tested positive for anti-IL-1Ra IgG in both Western blot (**B**) and ELISA (**Figure 3A**).


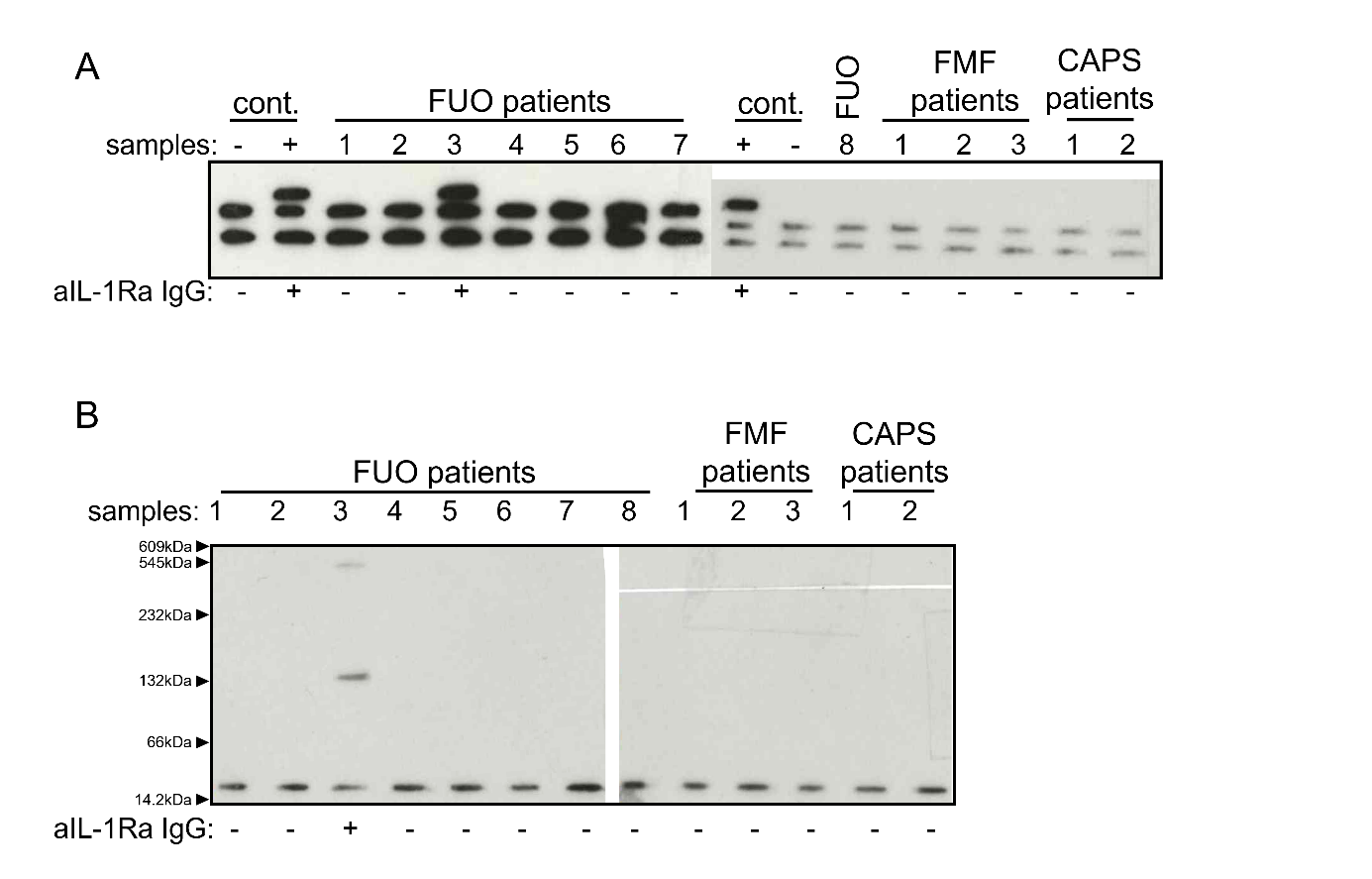


**Figure S5. IL-1Ra hyperphosphorylation and immune complexes in FUO, FMF and CAPS patients.** (A) Isoelectric focusing (IEF) of plasma IL-1Ra or (B) Western blot of native gradient PAGE for IL-1RA:IgG immune complexes. Plasma samples previously tested positive or negative for anti-IL-1Ra antibodies were used as positive or negative control (**A**). Seropositivity for anti-IL-1Ra IgG according to ELISA (**Figure S3**) is indicated by +/-.

**Figure S6. IL-36Ra immune complexes in Still’s disease patients’ plasma (cohort 2).** (A) Isoelectric focusing (IEF) of plasma IL-36Ra or (B) Western blot of native gradient PAGE for IL-36RA:IgG immune complexes in 35 Still’s disease patients (cohort 2). Plasma samples previously tested positive or negative for anti-IL-36Ra antibodies were used as positive or negative control. Seropositivity for anti-IL-36Ra IgG according to ELISA (**Figure 3A**) is indicated by +/-.

**Figure S7. Inflammatory mediators in anti-IL-1Ra seropositive *versus* seronegative Still’s disease serum or plasma samples.** (A) Luminex analysis of indicated markers in patients’ serum/plasma. (B-D) Proximity extension assay (Olink® Target 96 Inflammation Panel) data of a limited number of anti-IL-1Ra antibody positive sJIA *versus* seronegative sJIA and MIS-C serum samples. B) Heatmap based on raw npx (normalized protein expression) values of all acquired data. C) Selected markers with differential expression in seropositive versus seronegative sJIA samples. D) Identical markers as in B) but including seronegative MIS-C samples. A, C, D) Data were analyzed by Mann-Whitney U test. * = *P* <0.05; ** = *P* < 0.01

**Figure S8. IL-1b signaling reporter assay with additional patients’ plasma samples.** IL-1β signaling assay using HEK IL-1 reporter cells to evaluate the efficacy of anakinra (left) or canakinumab (right) to override the IL-1Ra depleting effect of anti-IL-1Ra positive Still’s disease plasma. TNFα, IL-36Ra and an anti-SLP2 antibody were used as negative controls in these assays. Cohort 1 plasma samples 20 (anti-IL-1Ra titer 1:400) and 10 (seronegative) were used at a dilution of 1:10. Data are presented as mean +/- SD, three replicates.

**References**

1. Thurner L, Preuss KD, Fadle N, Regitz E, Klemm P, Zaks M, et al. Progranulin antibodies in autoimmune diseases. J Autoimmun. 2013;42:29–38.

2. Grass S, Preuss KD, Ahlgrimm M, Fadle N, Regitz E, Pfoehler C, et al. Association of a dominantly inherited hyperphosphorylated paraprotein target with sporadic and familial multiple myeloma and monoclonal gammopathy of undetermined significance: a case-control study. Lancet Oncol. 2009 Oct;10(1474-5488 (Electronic)):950–6.
